# Impact of AMPK on cervical carcinoma progression and metastasis

**DOI:** 10.1038/s41419-023-05583-9

**Published:** 2023-01-19

**Authors:** Paweł Konieczny, Tomasz Adamus, Maciej Sułkowski, Klaudia Skrzypek, Marcin Majka

**Affiliations:** Jagiellonian University Medical College, Faculty of Medicine, Institute of Pediatrics, Department of Transplantation, Krakow, Poland

**Keywords:** Metastasis, Cancer models, Experimental models of disease, Cancer models

## Abstract

Cervical cancer (CC) is the fourth most common malignant neoplasm among women. Late diagnosis is directly associated with the incidence of metastatic disease and remarkably limits the effectiveness of conventional anticancer therapies at the advanced tumor stage. In this study, we investigated the role of 5’AMP-activated kinase (AMPK) in the metastatic progression of cervical cancer. Since the epithelial mesenchymal transition (EMT) is known as major mechanism enabling cancer cell metastasis, cell lines, which accurately represent this process, have been used as a research model. We used C-4I and HTB-35 cervical cancer cell lines representing distant stages of the disease, in which we genetically modified the expression of the AMPK catalytic subunit α. We have shown that tumor progression leads to metabolic deregulation which results in reduced expression and activity of AMPK. We also demonstrated that AMPK is related to the ability of cells to acquire invasive phenotype and potential for in vivo metastases, and its activity may inhibit these processes. Our findings support the hypothesis that AMPK is a promising therapeutic target and modulation of its expression and activity may improve the efficacy of cervical cancer treatment.

## Introduction

Cervical cancer (CC) is one of the most common malignant neoplasms in women [1]. CC mortality affects mainly women in developing countries and is particularly prevalent among patients with a low socio-economic status. An important factor contributing to high mortality is late diagnosis due to poor screening scheme. The main etiological factor of the disease is human papillomavirus (HPV) [2]. The development of a vaccine against the most oncogenic types of the virus (16 and 17, which account for 70% of all cases of CC) allows for the effective prevention of CC [3]. However, vaccination against HPV must be correlated with screening, which allows early detection of pre-cancer lesions. Early stages of the disease are relatively easily curable with conventional therapies such as chemotherapy and radiotherapy, but the chances of 5-year survival dramatically decrease with the presence of metastasis [4]. Thus, there is unmet need for new therapeutic approaches.

Epithelial to mesenchymal transition (EMT) is long-term morphological and molecular process, allowing cancer cells to lose cell-to-cell adhesion and acquire migratory and invasive properties [5, 6]. EMT has been demonstrated in pathological conditions such as organ fibrosis and metastatic progression of tumors [7] and it is similar to the physiological process of EMT during embryogenesis, although differences in many types of neoplasms are observed [8].

5’AMP-activated kinase (AMPK) is a cellular energy homeostasis sensor, that coordinates network of various metabolic pathways, such as cholesterol inhibition and fatty acid synthesis [9, 10]. At molecular level, AMPK controls balance between energy intake and demand, therefore modulating such processes as carbohydrate and lipid metabolism, biosynthesis, autophagy, and cell cycle [11]. Due to high energy demand during EMT process, several morphological and metabolic changes are orchestrated by AMPK.

Recently, AMPK has emerged as an important therapeutic target for anticancer therapies. Clinical study of type 2 diabetes patients treated with metformin (a pharmacological activator of AMPK), revealed lower cancer incidence rate comparing to the control group [12]. Moreover, metformin treatment demonstrated higher cancer remission rate in diabetic patients [13, 14]. In vitro studies confirmed the therapeutic potential of AMPK activation in many types of cancer [15, 16]. Interestingly, AMPK action in cancer progression may be related to blocking or even reversing the EMT.

Current knowledge does not allow unequivocally define the role of AMPK in tumor development and progression, often defining AMPK as a “double-edged sword”, whose action depends on the metabolic context [17, 18].

Here, we attempted to elucidate the contribution of AMPK to CC biology, particularly to EMT process and CC metastatic progression.

## Results

### Morphology and growth characteristics of cervical carcinoma cell lines

**Fig. 1:**
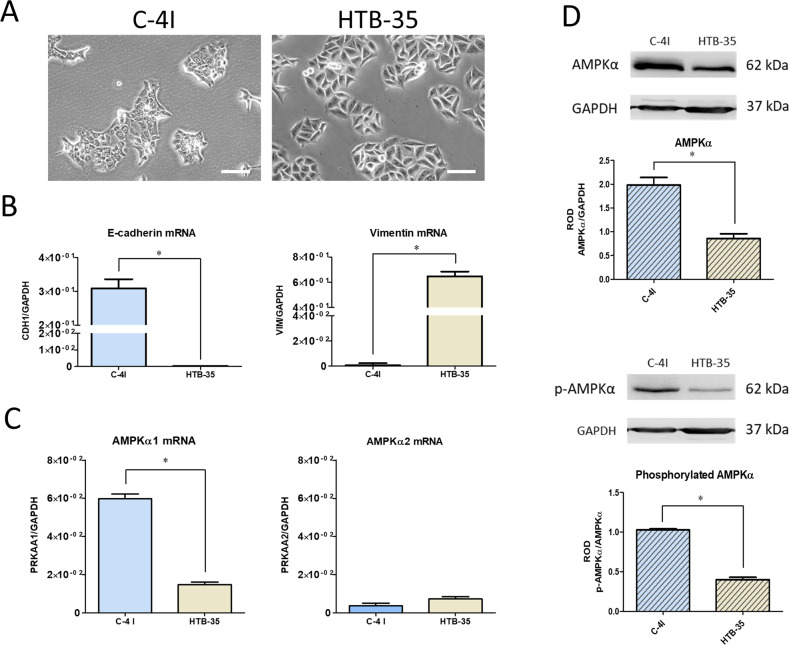
Characteristics of cervical carcinoma cell lines. Panel **A** presents the morphology of CC cells under normal growth conditions (white bar represents 100 µm). Expression of the major epithelial and mesenchymal markers (**B**) and *PRKAA1* (AMPKα1) and *PRKAA2* (AMPKα2) genes (**C**) assessed by qRT-PCR. The graphs represent the mean from at least three independent analyses of the relative expression of the gene to the reference gene GAPDH ± SEM. An asterisk indicates statistically significant differences (*p* < 0.05). Expression and activation of AMPKα protein (**D**) were evaluated using the Western Blot technique. Membrane pictures show representative results of three independent assessments. Quantitative results presented on bar graphs are based on densitometry of the analyzed gene to the reference gene. Data are presented as mean ± SEM (*n* = 3). Asterisks indicate statistically significant differences (*p* < 0.05).

Morphology and molecular properties of C-4I and HTB-35 cell lines represent different stages of tumor progression [19]. C-4I cells formed colonies with distinct boundaries, and cells were tightly adherent to each other, representing characteristic of epithelial cells, whereas HTB-35 cell line exhibited a loosen cell-to-cell junctions (Fig. 1A). Relative gene quantification showed differences in the expression of major markers associated with the EMT in these cell lines (Fig. 1B). E-cadherin (epithelial cell marker) was significantly decreased, while vimentin (mesenchymal cell marker) was expressed in abundance in HTB-35 cells. We concluded, that C-4I cell line recapitulates the properties of cells before EMT, while HTB-35 cells that underwent that process.

Next, we assessed AMPK expression level and activity. We showed higher expression level of the AMPKα1 catalytic subunit transcript in the C-4I cells (Fig. 1C). Expression of AMPKα2 subunit transcript was low in both cell lines. Importantly, there were also no differences in the amount of mRNA for β and γ subunits (data not shown). Higher expression of AMPKα in C-4I cells was confirmed by Western Blot (Fig. 1D). Detection of the phosphorylated form of the α subunit confirmed the higher activity of AMPK in C-4I cells (Fig. 1D).

### Effects of EMT-inducing factor HGF on AMPK expression and activation

**Fig. 2:**
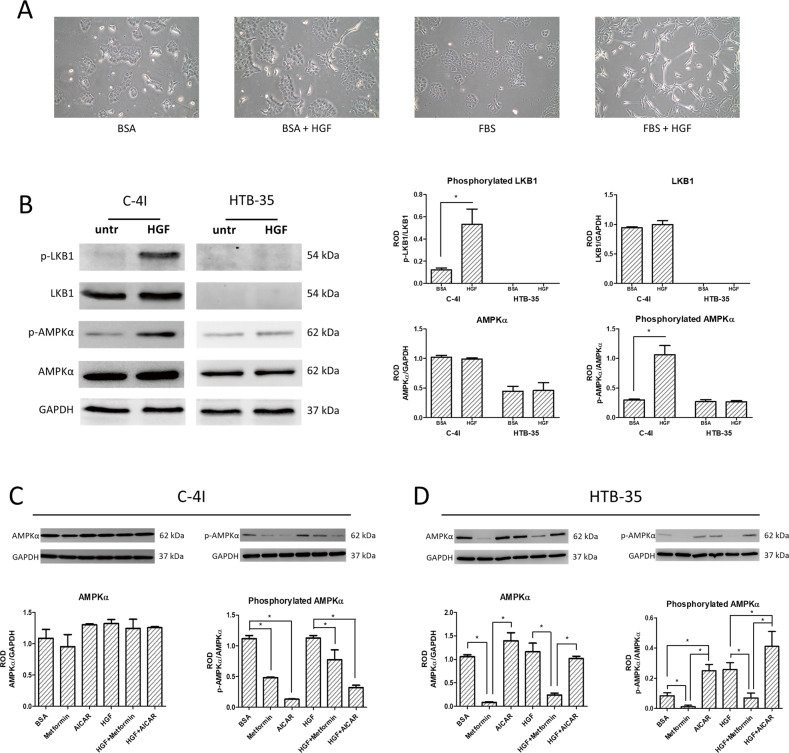
HGF factor influences phenotype of C-4I cells and expression of catalytic subunit AMPKα1. Observation of C-4I cell phenotype change after incubation with HGF, both in medium containing 0.5% BSA and 10% FBS (**A**) (white bar represents 100 µm). Evaluation of LKB1 and AMPKα expression and activation in C-4I and HTB-35 cells after 1 h HGF treatment (**B**). Expression and activation of AMPKα subunit after incubation with HGF and AMPK kinase activators (metformin and AICAR) in C-4I (**C**) and HTB-35 (**D**) cells after 24 h incubation. Membrane pictures show representative results of three independent experiments. Quantitative results presented on bar graphs are based on densitometry of the analyzed gene to the reference gene. Data are presented as mean ± SEM (*n* = 3). Asterisks indicate statistically significant differences (*p* < 0.05).

We and others showed previously that hepatocyte growth factor (HGF) is potent EMT inducer in cervical carcinoma [20, 21]. Thus, we tested the effect of HGF on the expression and activation of the AMPK in CC cells. 24 h incubation of C-4I cells with HGF induced a mesenchymal phenotype of the C-4I cells when cultured in 10% FBS (Fig. 2A). Assuming that the changes of cell shape alternate energy homeostasis, we examined if phenotypic changes were associated with AMPKα expression and activation. Expression of AMPKα1 transcript altered under EMT inducing conditions. The reduction in AMPKα1 expression was statistically significant after 24 h incubation with HGF, and further increased after 48 h (Supplementary Fig. S-1B). To investigate HGF impact on AMPKα activation we stimulated C-4I and HTB-35 cell lines with HGF and assessed phosphorylation of AMPKα. We noticed strong AMPKα activation in C-4I cells and virtually no changes in HTB-35 cells after 1 h stimulation (Fig. 2B). To examine that differences, we evaluated LKB1 expression and activation, which is an upstream kinase of AMPKα. We noticed lack of LKB1 expression in HTB-35 cells and phosphorylation of LKB1 and AMPKα in C-4I cell line (Fig. 2B).

After assessing the effect of HGF on the activation the AMPKα during 1 h treatment, we evaluated the changes in AMPK protein level after a 24 h incubation with HGF and pharmacological AMPK activators (Fig. 2C, D). We observed, that C-4I cells incubated with metformin and 5-Aminoimidazole-4-carboxamide ribonucleotide (AICAR) for 24 h lost the activity of the catalytic α subunit. Moreover, this effect was partially reversed by HGF treatment (Fig. 2C).

Surprisingly, we found that C-4I cells simultaneously incubated with HGF and AICAR, did not spread, nor exhibited phenotypic changes (Supplementary Fig. S-1A). Comparable, although weaker effect was observed when C4-I cells were incubated simultaneously with HGF and metformin. For HTB-35 cells, a 24 h incubation with metformin at a concentration of 10 mM led to partial cell death (Supplementary Fig. S-1C). Interestingly, a very low expression of AMPKα in these cells was found. In addition, virtually no phosphorylated form of AMPKα was detected in cells treated with metformin (Fig. 2D).

### Changes in the expression and activation of the AMPK catalytic subunit α and impact on the expression of markers associated with the EMT

**Fig. 3:**
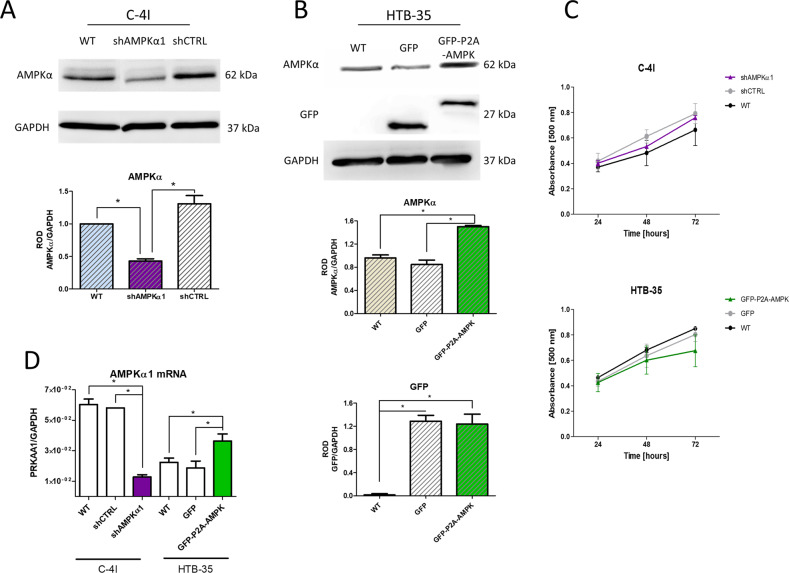
Generation of genetically modified CC lines with increased and decreased AMPKα expression. The evaluation of AMPKα1 catalytic subunit knockdown in C-4I cells (**A**) and GFP and AMPKα protein expression in selected and sorted HTB-35 lines (**B**). Membrane pictures show representative results of three independent assessments. Quantitative results presented on bar graphs are based on densitometry of the analyzed gene to the reference gene. Data are presented as mean ± SEM (*n* = 3). Asterisks indicate statistically significant differences (*p* < 0.05). Determination of cell proliferation of modified C-4I and HTB-35 lines by MTS (**C**). The points on the graph show the mean absorbance of three replicates ± SEM. **D** Transcript level of AMPKα1 catalytic subunit in 6 lines of the developed cellular model. The graphs represent the mean of three independent analyses of the relative expression of the gene to the reference gene GAPDH ± SEM. Asterisks indicate statistically significant differences (*p* < 0.05).

Genetic modifications were used to silence the AMPKα1 subunit in C-4I cells and to upregulate the expression of the AMPKα1 subunit in HTB-35 cell line. To achieve this, we knocked down AMPK subunit α1 expression in C-4I cells using lentiviral particles introducing shPRKAA1 (shAMPKα1). We upregulated the expression of AMPKα1 in HTB-35 cells by introducing GFP-P2A-PRKAA1 (GFP-P2A-AMPK) transgene using lentiviral vectors. We verified the downregulation and overexpression of AMPKα1 mRNA level in genetically modified C-4I and HTB-35 cell lines (Fig. 3D) as well as the protein level of AMPKα1 (Fig. 3A, B). The proliferation of genetically modified CC cells showed no statistically significant changes. Regardless, partial decrease in the growth rate of HTB-35 GFP-P2A-AMPK line cells was observed (Fig. 3C).

**Fig. 4:**
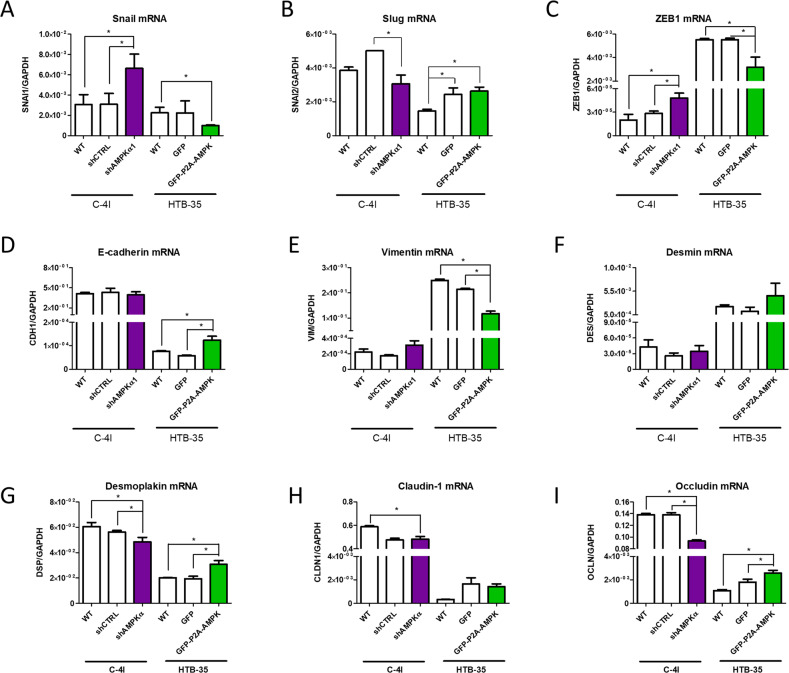
Modification of AMPKα1 catalytic subunit expression in the CC cells alternates the expression of EMT-related factors. Evaluation of expression of transcription factors associated with the induction of epithelial to mesenchymal transition (**A**–**C**) and transcript level of epithelial and mesenchymal phenotype markers (**D**–**F**). Transcript level of proteins involved in cell-to-cell connections (**G**–**I**). The graphs represent the mean of three independent evaluations of the relative expression of the analyzed gene to the reference gene GAPDH ± SEM. Asterisks indicate statistically significant differences (*p* < 0.05).

Engineered CC cell lines with modified AMPKα expression served as a model to evaluate the expression of markers associated with EMT. We assessed the expression of transcription factors (TFs), as well as changes in mesenchymal phenotype markers or other factors related to EMT process such as intercellular junction proteins (Fig. 4A–I).

We discovered significant differences in EMT-related transcription factors in cell lines with altered AMPKα expression. In particular, we observed changes in SNAIL and ZEB-1 TFs (well-known ‘triggers’ of EMT process) as their expression correlated negatively with AMPKα expression in both C-4I and HTB-35 lines (Fig. 4A, C). C4-I shAMPKa1 cells showed elevated levels of SNAIL and ZEB-1, whereas the opposite effect was observed in the HTB-35 GFP-P2A-AMPK. Elevated AMPKα1 expression in the HTB-35 line correlated with lower expression of the vimentin and higher of E-cadherin, indicating re-establishment of cells epithelial properties (Fig. 4D, E). Interestingly, no significant changes in the expression of the E-cadherin nor the vimentin were found in C-4I cell lines.

Our analysis revealed changes in the mRNA expression of the intercellular junction proteins as well. Significant differences in the expression of desmoplakin and occludin were in line with changes of major epithelial and mesenchymal markers (E-cadherin and vimentin). In particular, knockdown of AMPKα decreased the expression of desmoplakin and occludin in C-4I shAMPKα1 cells (Fig. 4G, I). Again, the opposite effect was observed for HTB-35 cell lines, as AMPKα overexpression resulted in the downregulation of junction proteins.

**Fig. 5:**
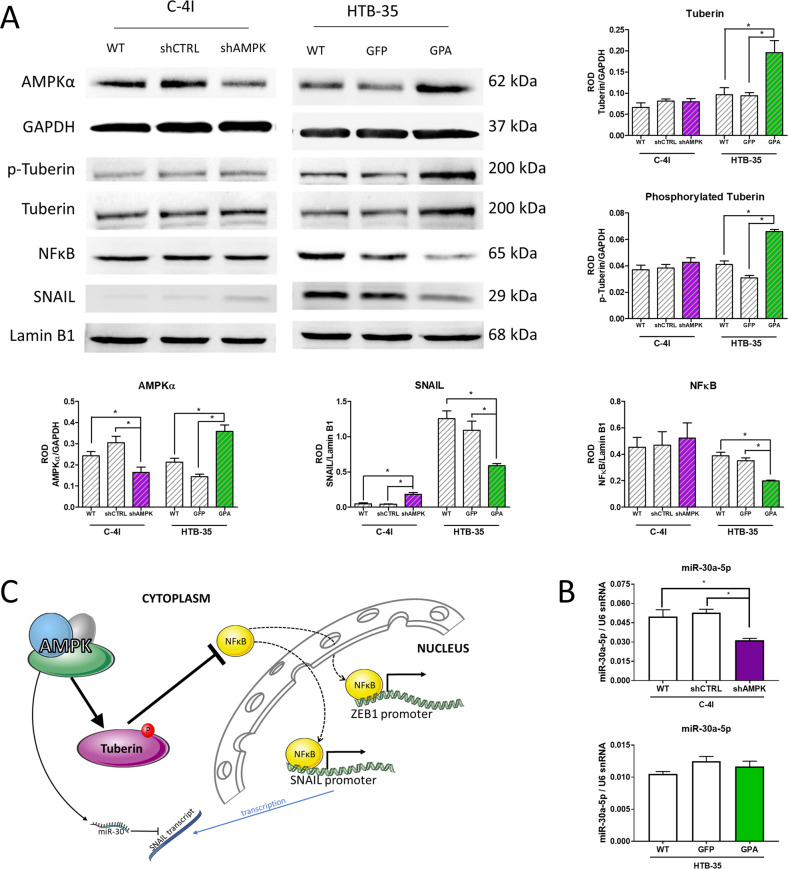
Modification of AMPKα1 expression in the CC cells moderates the expression of Snail and ZEB-1 via tuberin-NFκB pathway. **A** Changes in tuberin expression and activation, NFκB, and SNAIL expresion as a result of AMPKα expression modification; **B** evaluation of expression levels of miR-30a-5p in C-4I and HTB-35 modified cells. **C** Scheme of AMPK regulation of SNAIL and ZEB1 expression; Membrane pictures show representative results of three independent experiments. The graphs represent the mean of three independent evaluations of the relative expression of the analyzed gene to the reference gene ± SEM. Asterisks indicate statistically significant differences (*p* < 0.05).

To further investigate changes in EMT-related transcription factors after AMPKα expression modification, we examined the expression and activation level of tuberin (known tumor suppressor associated with AMPK as well as AKT and mTOR [22]). We revealed that in HTB-35 GFP-P2A-AMPK (GPA) cell line tuberin level was elevated along with its phosphorylation. Higher tuberin level was associated with downregulation of NFκB and reduced level of SNAIL transcription factor. Opposite effect was expected for C-4I shAMPKa1 cells, however, we did not find such regulation (Fig. 5A). It is possible that in C-4I cell line, AMPKα1 regulates SNAIL by miR-30 family. We observed that AMPKα1 silencing diminished the levels of miR-30a-5p (Fig. 5B) and also different members of miR-30 family: miR-30b-5p, miR-30c-5p, miR-30d-5p, miR-30e-5p and miR-30a-3p (Supplementary Fig. 4). Contrary to that, we did not find alterations in miR-30 family in HTB-35 cells (Fig. 5B and Supplementary Fig. S–4). We summarized the AMPK regulatory mechanisms in Fig. 5C.

### Tumor growth and metastatic abilities of C-4I and HTB-35 cell lines

**Fig. 6:**
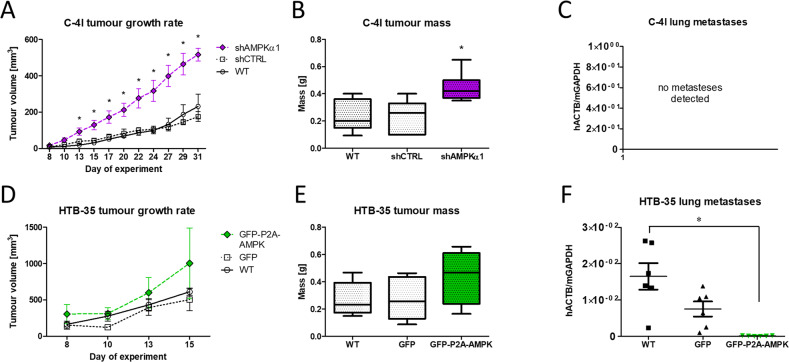
Tumor formation by subcutaneous injection of CC cells in the NOD-SCID mice. The volumetric growth rate of tumors calculated from percutaneous tumor measurements for C-4I (**A**) and HTB-35 (**D**) cells. Box plots show the mass of isolated tumors for each of three lines C-4I (**B**) and HTB-35 (**E**). Assessment of human β-actin transcript levels in lung samples isolated from mice with C-4I and HTB-35 injected cells (**C**, **F**). The ability to form metastases is directly proportional to the amount of human β-actin transcript in relation to the mouse GAPDH gene. All graphs represent data from two rounds of independent experiments ± SEM (2 × *n* = 3 for each cell line). Asterisks indicate statistically significant differences (*p* < 0.05).

We aimed to verify the influence of AMPKα expression on metastatic capacity of the cells in NOD-SCID mice. C-4I cells formed tumors when injected subcutaneously into mice (Figs. 6A and S-5). However, C4-I shAMPK cells exhibited significantly faster tumor growth than controls, what was corroborated with tumor weight assessed in the end of experiment (Fig. 6B). Despite that, no human β-actin gene transcript was detected in RNA samples collected from mice injected with C-4I cells (Fig. 6C). This indicated the absence of metastasis formation, regardless of AMPK expression.

We implemented the same experimental approach for HTB-35 cell lines. We found no statistically significant changes in tumor growth and weight (Fig. 6D, E). The ability of HTB-35 cells to form metastases was assessed by RT-qPCR. Analysis of RNA extracted from lung, spleen and marrow specimens revealed the presence of human β-actin gene transcripts in lungs, indicating HTB-35 cells metastasis. Interestingly, the amount of human RNA in the lungs of mice injected with GFP-P2A-AMPK cells was significantly lower in comparison with control groups (Fig. 6F) indicating lower metastatic potential.

## Discussion

The main goal of this study was to correlate the AMPK expression and activation with the acquisition of invasive capabilities through the implementation of EMT.

Our findings revealed that AMPK expression is related to malignant behavior of CC cells. In C-4I line, AMPKα expression and activation stays at a higher level than in HTB-35 cells. Various literature reports show restrained AMPKα expression and activity in multiple types of tumors. Chi-Wai Lee et al. demonstrated a decrease in AMPK expression in different liver cancer cell lines [23]. The authors showed that the suppression of AMPKα2 expression in HepG2 cells was associated with an epigenetic modification in the *PRKAA2* gene promoter region (encoding AMPKα2). The assessments carried out by Hadad et al. on breast cancer histology samples allowed to correlate the reduced AMPK activity with the development of the tumor [24]. In all cases, significantly lower AMPKα activity was determined in tumor tissues than in the surrounding normal epithelial tissue. Additionally, the authors demonstrated that the AMPKα activity is positively correlated with tumor malignant properties. Similar conclusions were reached by researchers analyzing samples of pancreatic cancer tumors [25]. IHC staining revealed that AMPKα in cancer cells was deactivated, as opposed to normal epithelium. All above support our hypothesis, that the acquisition of invasive capacity and increase of malignancy rate are positively correlated with the expression and activity of AMPK (especially AMPKα) in CC cells.

The induction of EMT by HGF is well described [20, 26, 27]. To analyze the influence of HGF on AMPK, the expression and activation of AMPKα was determined. Short time incubation with HGF resulted in activation of AMPKα only in C-4I cell line. As shown by Vázquez-Chantada et al. and Esteve-Puig, HGF is the factor that triggers AMPK *via* LKB1 [28, 29]. Interestingly, no activation was visible in HTB-35 cells. As it turned out, that was due to the lack of functional LKB1 in those cells. The absence of LKB1 in HTB-35 cell line is caused by homozygous deletion of STK11/LKB1 locus [30]. Thus, we can assume that rapid activation of AMPK by HGF (for a short time treatment) solely depends on LKB1. In contrast, long time incubation with HGF may be more complex and involve other pathways than LKB1, as we could notice AMPKα phosphorylation in HTB-35 cells treated with HGF for 24 h. Moreover, in C-4I cell line, long incubation with HGF (in the presence of 10% FBS) led to phenotypic changes and scattering effect, but the addition of the AMPK activator AICAR completely blocked this effect. Similar but weaker effect was also evident with simultaneous incubation of HGF with either metformin or medium with low glucose concentration, therefore AMPK activation led to inhibition of cell dispersal. We found, that in C-4I cells incubated with metformin and AICAR for 24 h, the activation of AMPKα was diminished. Additional experiments are necessary to fully address this observation. Nevertheless, AMPK activation triggered by HGF remained at a high level but simultaneous incubation with HGF and AMPK activators resulted in lower activation than HGF alone. Remarkably, after 24 h of incubation in the medium with 0.5% BSA (without any additional factors added), AMPKα appeared to be activated due to induced nutrient deficiency in the medium and natural activation [31].

We could explain observed phenomena by the fact that C-4I cells, as retaining some features of normal epithelial cells, also maintain a metabolic ‘regime’. This means that long-term and non-physiological activation of the AMPK will be unfavorable for them because the AMPK loses its proper function, that is the metabolic switch—continuous activation makes it impossible to react to changes. Therefore, the cells switch off the AMPK activity. However, the influence of HGF bypasses this mechanism and leads to permanent activation of AMPKα, probably by changing the concentration of Ca^2+^ ions and activation of CaMKK [32, 33]. Hence, incubation with HGF (in a medium with 10% FBS) leads to the re-arrangement of the cytoskeleton and the acquisition of invasive phenotype, as energy is still supplied by AMPK. In turn, the observed silencing of AMPK activity in case of simultaneous incubation with AICAR and HGF explains the inhibition of cell dispersion by switching the AMPK from energy generation to energy storage processes.

AMPK activation by HGF and pharmacological AMPK activators in HTB-35 cells appears to be completely different than in C-4I cells. It is possible that we observed an active mechanism degrading the catalytic subunit of AMPKα. Studies conducted by Pineda et al. showed that MAGE-A3/6-TRIM28 ubiquitous ligase caused ubiquitination and subsequent degradation of α1 subunit in different types of neoplasms [34]. Importantly, it was associated with the hypersensitivity of cancer cells to AMPK activator— metformin. The occurrence of this mechanism in HTB-35 cells could explain their high sensitivity to long-term incubation with metformin. We also observed that effect for AICAR-treated cells, however, it was visible after 48 to 72 h of incubation.

We illustrated the dual nature of AMPK in examined CC cells, most likely resulting from differences in cell invasiveness and malignancy [35]. HTB-35 cells utilize AMPK not as a “guardian” of metabolism, but as a “helper” in gaining energy, e.g., through disorders in lipid metabolism. However, the use of metformin, whose action is less selective than AICAR, in HTB-35 cells leads to a decrease in the efficiency of energy acquisition in the process of glycolysis and, consequently, cell death [36].

We noticed that different levels of AMPK expression led to different expression levels of intercellular junction proteins. RNA expression for desmoplakin and occludin was elevated in HTB-35 cells with AMPKα overexpression. Noteworthy, AMPKα knockdown in C-4I cells resulted in exactly opposite effect – lowered transcript level of intercellular junction proteins was detected. These observations underline AMPK impact on phenotype change during EMT. Moreover, we conclude that the reduction of AMPK expression is correlated with the first step for the acquisition of motility properties. The changes in AMPK expression have also an impact on transcription factors associated with EMT. AMPKα1 knockdown in C-4I cells increased the expression of SNAIL and ZEB-1 factors, while increased AMPKα1 expression in HTB-35 cells led to decrease levels of these TFs. Our experiments revealed relation between AMPK and SNAIL *via* tuberin-NFκB pathway in HTB-35 cells in the similar manner as described by Liang et al. [37]. Moreover, downregulation of NFκB can affect ZEB-1 expression as well, since NFκB is able to bind to ZEB-1 promoter [38]. Thus, NFκB downregulation can reduce SNAIL and ZEB-1 transcriptional activity. Interestingly, we did not find such regulation in C-4I cell line. In C-4I cells, different mechanism of AMPK action may be involved. In that cell line, AMPK regulates SNAIL probably by members of miR-30 family. AMPK knockdown has been previously associated with downregulation of miR-30a [39] or miR-30b [40]. miR-30a-5p targets SNAIL transcription factor and diminishes its levels [41]. The other members of miR-30 family may also regulate SNAIL mRNA levels [42]. On the other hand, no regulation of SNAIL *via* miR-30 family was found in HTB-35 cells. Different types of SNAIL expression modulation by AMPK in tested cell lines may relate to their different invasive properties. Considering above, we believe that these mechanisms may be active during cervical cancer progression (Fig. 5C). HTB-35 cell line overexpressing AMPKα displays differences in the expression of the main EMT markers, such as vimentin and E-cadherin, showing a partial reversal of mesenchymal phenotype. C-4I shAMPKα1 cell line, apart from changes in the expression of transcription factors, did not show significant differences in E-cadherin or vimentin. This suggests that the decrease in AMPK expression alone is not sufficient to trigger changes in the EMT phenotype. This is consistent with the literature reports, connecting the reduction of this kinase activity with the acquisition of invasive capacity by cancer cells [43].

Although we did not notice significant changes in the growth rate between unmodified and modified cells in vitro, AMPKα knockdown in C-4I cells enhanced their ability to grow in vivo. After transplantation to the NOD-SCID mouse model, C4-I shAMPKa1 cells formed larger and faster growing tumors than control cells. This is consistent with the published data, which correlates the expression of AMPKα directly with the tumor growth rate in the mouse model [44]. However, the silencing of AMPKα expression did not affect the lack of ability of C-4I cells to metastasize. Considering the lack of significant changes of EMT major markers (E-cadherin and vimentin) in these cells, and the fact that EMT is the necessary mechanism for the metastasis in CC [45], this result is not surprising.

Conversely, an increase of AMPKα1 expression in HTB-35 cells, which was associated with a partial reversal of the mesenchymal phenotype in vitro, limited the ability of these cells to metastasize in vivo. Considering the fact that this kinase is natively active in HTB-35 cells (although at a low level), the increase in the amount of AMPKα protein will simultaneously result in proportional increase in its activity. It is in accordance with most of the publications, indicating that AMPK activation leads to a decrease metastatic capability [25, 46].

AMPK can take on different functions depending on the molecular and metabolic context [47, 48]. We showed here that lowering the expression of AMPKα in C-4I cells and overexpression in HTB-35 cells lead to the promotion of cells survival in vivo. This may be due to the different function of AMPK in these cells. In C-4I line, which retains some of the properties of normal epithelial tissue, AMPK still plays the correct role of metabolic “guardian”, whose exclusion leads to metabolic deregulation and acquisition of a higher degree of malignancy. In HTB-35 cells, there is a permanent decoupling of metabolic pathways, therefore AMPK falls out of its function. HTB-35 cells, which acquire energy mainly in the process of glycolysis [49], can use AMPK only to increase the ability to generate energy, without metabolic regulation [50].

Dissecting the role of AMPK in regulating many intracellular processes is still a demanding issue. Despite the fact that AMPK may play a double role in the cancer progression, it is a promising therapeutic target. Especially in the early stages of CC, the AMPK activators may benefit in favorable therapeutic outcome. In the advanced neoplastic disease, or to be more precise, in the case of deregulation the expression and activation of AMPK in CC cells, the restoration of AMPK expression may be a step towards the elimination of cancer cells.

## Material and methods

### Cell culture conditions

Cell lines were obtained from ATCC and routinely tested for mycoplasma contamination (MycoAlert® PLUS Mycoplasma Detection Kit, Lonza, Switzerland). C-4I and HTB-35 cell lines were maintained as a monolayer cultures in Waymouth’s medium (Thermo Fisher Scientific, MA, USA) and EMEM medium (Lonza, Switzerland), respectively, supplemented with 10% v/v fetal bovine serum (FBS; EURx, Poland), and 100 U/ml penicillin and 100 µg/ml streptomycin (both from Thermo Fisher Scientific, Waltham, MA, USA). Cells were cultured at 37 °C in a humidified atmosphere of 5% CO_2_.

### Genetic modification of C-4I and HTB-35 cells

Cloning and lentivirus production was previously described in ref. [51]. Further details of modified cell lines generation are described in Supplementary Materials.

### qRT-PCR

Total RNA was isolated with GeneMATRIX Universal RNA/miRNA kit (Eurx, Poland), followed by reverse transcription (RT) using M-MLV reverse transcription kit (Promega, Madison, WI, USA). Blank qPCR Master Mix kit (EURx, Poland) with specific TaqMan probes (Thermo Fisher Scientific, USA) were used (Table 1). Reverse transcription of miRNA was performed using the NCode VILO miRNA cDNA Synthesis Kit (Invitrogen), according to the manufacturer’s protocol. For the evaluation of miRNA expression by quantitative real-time PCR, SYBR Green qPCR Master Mix (EURx) and universal reverse primer from the NCode VILO miRNA cDNA Synthesis Kit (Invitrogen) were used with the indicated forward primers designed according to the kit’s instruction (primers are listed in Supplementary materials). The ΔCt method (2^-∆Ct^) was used to calculate relative expression of the genes, using GAPDH as a relative control for mRNA and U6 snRNA as a control for miRNA.

**Table 1:** Taq-Man qRT-PCR probes list.

Gene name	Cat no.	Gene name	Cat no.
PRKAA1	Hs01562315_m1	VIM	Hs00958111_m1
PRKAA2	Hs00178903_m1	GFP	Mr03989638_mr
PRKAB1	Hs00272166_m1	DSP	Hs00950591_m1
PRKAG1	Hs01091629_g1	CLDN1	Hs00221623_m1
SNAI1	Hs00195591_m1	OCLN	Hs00170162_m1
SNAI2	Hs00161904_m1	DES	Hs00157258_m1
CDH1	Hs01023894_m1	MMP-2	Hs00234422_m1
ZEB-1	Hs00232783_m1	ACTB	Hs99999903_m1
MMP-1	Hs00899658_m1	MMP-13	Hs00233992_m1
mGAPDH	Mm99999915_g1	hGAPDH	Hs02786624_g1

### Western Blot analysis

The total protein fraction was extracted using M-PER buffer (Thermo Fisher Scientific, USA) with protease and phosphatase inhibitors (Sigma Aldrich, USA). The protein concentration was determined by Bradford method. After SDS-PAGE and proteins transferred on PVDF membranes were incubated overnight at 4 °C with primary antibodies (Table 2), and subsequently detected with HRP-conjugated goat anti-rabbit IgG secondary antibody (1:4000; Santa Cruz Biotechnology, USA). The membranes were developed with SuperSignal West Pico Chemiluminescence Substrate (Thermo Fisher Scientific, USA) with Gel Logic Imaging System (Kodak, USA). Pictures of full uncropped membranes can be found in ‘Supplementary materials uncropped WBs’.

**Table 2:** Antibodies utilized for protein detection.

Antibody target	Manufacturer	Cat no
AMPKα	Cell Signaling	#2793
Phospho-AMPKα	Cell Signaling	#4188
AMPKβ1	Cell Signaling	#4178
Phospo-AMPKβ1	Cell Signaling	#4186
GFP	Cell Signaling	#2955
GAPDH	Cell Signaling	#2118
goat anti-rabbit IgG-HRP	Santa Cruz Biotechnology	sc-2004
goat anti-mouse IgG-HRP	Santa Cruz Biotechnology	sc-2005
LKB1	Santa Cruz Biotechnology	sc-32245
Phospho-LKB1	Cell Signaling	#3482
Tuberin	Cell Signaling	#4308
Phospho-Tuberin	Cell Signaling	#23402
NFκB	Cell Signaling	#8242
SNAIL	Cell Signaling	#3895

### MTS proliferation assay

The CellTiter 96® AQueous One Solution Cell Proliferation Assay kit (Promega, USA) was used to assess the proliferation of the CC cells. Cells were seeded into 96-well plates - the number of cells seeded was 1 × 10^4^ and 5 × 10^3^ for C-4I and HTB-35 cell lines, respectively. The assay was performed at three time points: 24, 48, and 72 h after cell seeding.

### Xenografts in NOD-SCID mouse model

Animal experiments were conducted in accordance to ethical committee guidelines with approval of Local Institutional Animal Care and Use Committee (IACUC) in Krakow. Mice were randomly divided into six groups (*n* = 6 for each group—minimal group size that allowed to observe statistically significant differences and to meet Three Rs principle). 2 × 10^6^ C-4I cells (WT, shCTRL and shAMPKα1) or 1 × 10^6^ HTB-35 (WT, GFP, GFP-P2A-AMPK) were injected in 200 μL of PBS/growth factor-reduced Matrigel (Corning) 1:1 into left dorsal flank of adult (6–8 weeks old) female NOD/SCID mice. When tumor became palpable, its size was measured every 2–3 days. Experiments were terminated when tumor volume exceeded 1000 mm^3^ or earlier based on animal welfare. Mice were sacrificed, tumors, bone marrow, spleen, liver, and lungs were collected and frozen in LN2. Prior to RNA extraction, the frozen tissue fragments were mechanically homogenized in a Tissuelyser II device (Qiagen, Germany) and total RNA was isolated with GeneMATRIX Universal RNA Purification Kit (EURx, Poland).

### Statistical analysis

Statistical analysis was performed using GraphPad Prism 5. If not stated differently, presented data represent three independent experiments. For comparisons of two groups of data, the Student t test was used. For comparisons of multiple groups, analysis of variance one-way ANOVA with Tukey’s post hoc test was used. The Brown–Forsythe test was used to compare similarity of variances. If not stated otherwise, normal distribution was assumed. Graphs present mean ± SEM. Results with p smaller than *p* = 0.05 were considered as statistically significant.

## Supplementary information

Supplementary materials

Supplementary materials uncropped WBs

Reproducibility checklist

Authorship confirmation

## Data Availability

All datasets generated and analyzed during this study are included in this published article and its Supplementary Materials. Additional data are available from the corresponding author on reasonable request.
