# Supplementary figures and images for: Impact of AMPK on cervical carcinoma progression and metastasis
Source: Cell Death Dis. 2023 Jan 19;14(1):43. doi: 10.1038/s41419-023-05583-9 (PMC9852279; doi:10.1038/s41419-023-05583-9)

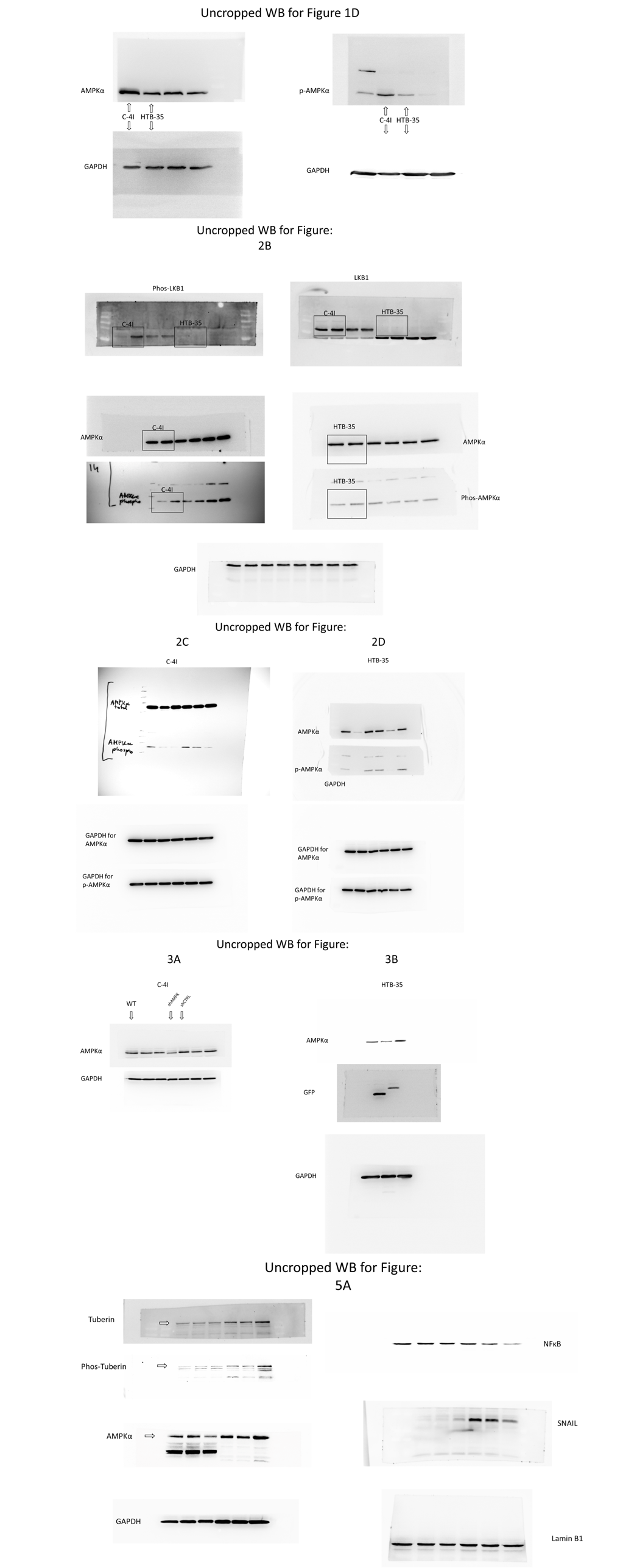

Supplement: Supplementary file 2 — Supplementary materials uncropped WBs [file 41419_2023_5583_MOESM2_ESM.tif]
