# Supplementary material for: Impact of AMPK on cervical carcinoma progression and metastasis
Source: Cell Death Dis. 2023 Jan 19;14(1):43. doi: 10.1038/s41419-023-05583-9 (PMC9852279; doi:10.1038/s41419-023-05583-9)
Supplement: Supplementary file 4 — Authorship confirmation [file 41419_2023_5583_MOESM4_ESM.pdf]

Temat **Authorship confirmation**
Od Paweł Konieczny <p.konieczny@uj.edu.pl>
Do Marcin Majka <mmajka@cm-uj.krakow.pl>
Data 2022-12-05 16:01

roundcube
open source webmail software

To whom it may concern,

As a co-author of manuscript entitled *Impact of AMPK on cervical carcinoma progression and metastasis* (CDDIS-22-0917R), I accept the change in authorship and confirm Dr Klaudia Skrzypek as co-author, regarding her contribution to its final form.

Pawel Konieczny

Od: Tomasz Adamus
Wysłano: środa, 30 listopada 2022 03:16
Do: Paweł Konieczny
Temat: Authorship statement

To whom it may concern,

As a co-author of manuscript entitled *Impact of AMPK on cervical carcinoma progression and metastasis* (CDDIS-22-0917R), I accept the change in authorship and confirm Dr Klaudia Skrzypek as co-author, regarding her contribution to its final form.

Best regards,
Tomasz Adamus

Od: Maciej Sułkowski
Wysłano: poniedziałek, 28 listopada 2022 10:16
Do: Paweł Konieczny
Temat: Authorship confirmation

As a co-author of the manuscript entitled 'Impact of AMPK on cervical carcinoma progression and metastasis' (CDDIS-22-0917R) I accept the change in authorship and confirm Dr Klaudia Skrzypek as co-author.

Sincerely,
Maciej Sułkowski

Od: Klaudia Skrzypek
Wysłano: poniedziałek, 9 stycznia 2023 09:26
Do: Marcin Majka; Paweł Konieczny
Temat: confirmation of authorship

To whom it may concern,

As a co-author of manuscript entitled *Impact of AMPK on cervical carcinoma progression and metastasis* (CDDIS-22-0917R), I accept my authorship, as described in the final revised version of the manuscript.

Klaudia Skrzypek

Klaudia Skrzypek, PhD
Department of Transplantation
Jagiellonian University Medical College
Wielicka 265
30-663 Krakow, Poland
phone: +48 12 6591593

Temat	**confirmation**
Od	Marcin Majka <mmajka@cm-uj.krakow.pl>
Do	'Marcin Majka' <mmajka@cm-uj.krakow.pl>
Data	2023-01-09 08:25

roundcube
open source webmail software

To whom it may concern,

As a co-author of manuscript entitled Impact of AMPK on cervical carcinoma progression and metastasis (CDDIS-22-0917R), I accept the change in authorship and confirm Dr Klaudia Skrzypek as co-author, regarding her contribution to its final form.

Marcin Majka
